# Inflammatory pseudotumor of Castleman disease and IgG4-related disease masquerading as kidney malignancy

**DOI:** 10.1186/s13000-021-01134-y

**Published:** 2021-08-10

**Authors:** Bolong Liu, Yong Huang, Luying Tang, Jiexia Guan, Xiangfu Zhou, Hailun Zhan

**Affiliations:** 1 Department of Urology, the Third Affiliated Hospital of Sun Yat-Sen University, Guangzhou, China; 2 Department of Pathology, The Third Affiliated Hospital of Sun Yat-Sen University, Guangzhou, China

**Keywords:** Kidney malignancy, Castleman disease, IgG4-related disease, Inflammatory pseudotumor

## Abstract

**Background:**

With widespread clinical application of imaging techniques, renal space-occupying lesions have been identified at an increasing frequency. Here, we report two rare cases, Castleman disease (CD) and IgG4-related disease (IgG4-RD), presenting primarily with the symptoms and imaging findings of kidney malignancy.

**Case presentation:**

In case 1, an occupying lesion located in the right renal pelvis was detected using magnetic resonance imaging in a 32-year-old female who presented with hematuria and lumbago. First misdiagnosed as carcinoma of the renal pelvis, the patient underwent right radical nephroureterectomy. However, postoperative pathological and immunohistochemistry studies finally confirmed the diagnosis of CD. In case 2, a 45-year-old male presented with the chief complaint of anuria. Nephrostomy and renal biopsy indicated lymphoma, following which, antegrade urography and computed tomography urography were performed, which revealed bilateral hydronephrosis and mass lesions around the renal pelvis. Partial resection of the masses and frozen section examination indicated the diagnosis of CD. However, the results of postoperative histopathology and immunohistochemistry combined with serum IgG4 were consistent with IgG4-RD. Both the patients recovered well after drug treatment without recurrence of the diseases.

**Conclusions:**

Inflammatory pseudotumor of CD and IgG4-RD with kidney involvement are primarily diagnosed by postoperative histopathology and can pose a preoperative diagnostic challenge because these lesions can masquerade as kidney malignancy. Therefore, we recommend core biopsy as a nonnegligible procedure to evaluate renal masses and potentially prevent unnecessary surgical treatment.

## Background

With widespread clinical application of computed tomography (CT), magnetic resonance imaging (MRI), ultrasound, and other imaging technologies, renal space-occupying lesions have been detected at an increased frequency. Renal space-occupying lesions include common diseases of the urinary system. The most frequently encountered malignant renal masses in clinical practice are renal cell carcinoma, urothelial carcinoma, lymphoma, and metastasis, while the most common benign renal solid masses include angiomyolipoma, renal oncocytoma, and inflammatory pseudotumor [1]. However, making preoperative diagnoses remains a challenge owing to rare benign masses with atypical imaging findings, which is often confused with kidney malignancy. Among numerous highly unusual diseases, inflammatory pseudotumor of Castleman disease (CD) and IgG4-related disease (IgG4-RD) are often misdiagnosed by clinicians as malignant neoplasms of the kidney. This can be attributed to their lack of typical or specific clinical manifestations and imaging characteristics, and their ability to manifest as renal lesions [2, 3].

CD is a rare lymphoproliferative disorder, primarily presenting as regional or generalized lymphadenopathy with multiorgan involvement. The disease can be classified into three histological variants, namely, hyaline vascular (HV) type, plasma cell (PC) type and a mixed form [4, 5]. IgG4-RD is an emerging idiopathic immune-mediated disorder characterized by elevated serum IgG4 concentration and the presence of IgG4-bearing lymphoplasmacytic infiltrates, resulting in chronic progressive inflammation accompanied by fibrosis and sclerosis in the affected organs [6, 7]. Both diseases present with a broad spectrum of common clinical manifestations, with diagnosis principally dependent on pathological examination. Moreover, CD and IgG4-RD exhibit partially overlapping clinical and pathological features, as lymphadenopathy, infiltration by IgG4-positive plasma cells, and enhanced IgG4 levels in serum, complicating diagnosis of these conditions [8]. Inflammatory pseudotumor of CD or IgG4-RD, masquerading as kidney malignancy, can lead to inappropriate therapy or overtreatment, and may aggravate patients’ conditions. Therefore, differential diagnosis of CD and IgG4-RD from kidney malignancy based on clinical manifestations, imaging, and pathological findings is essential. Here, we report one case each of CD and IgG4-RD, presenting primarily with symptoms and imaging findings of kidney malignancy, admitted to our hospital between 2016 and 2019.

## Case presentation

### Case 1

**Fig. 1:**
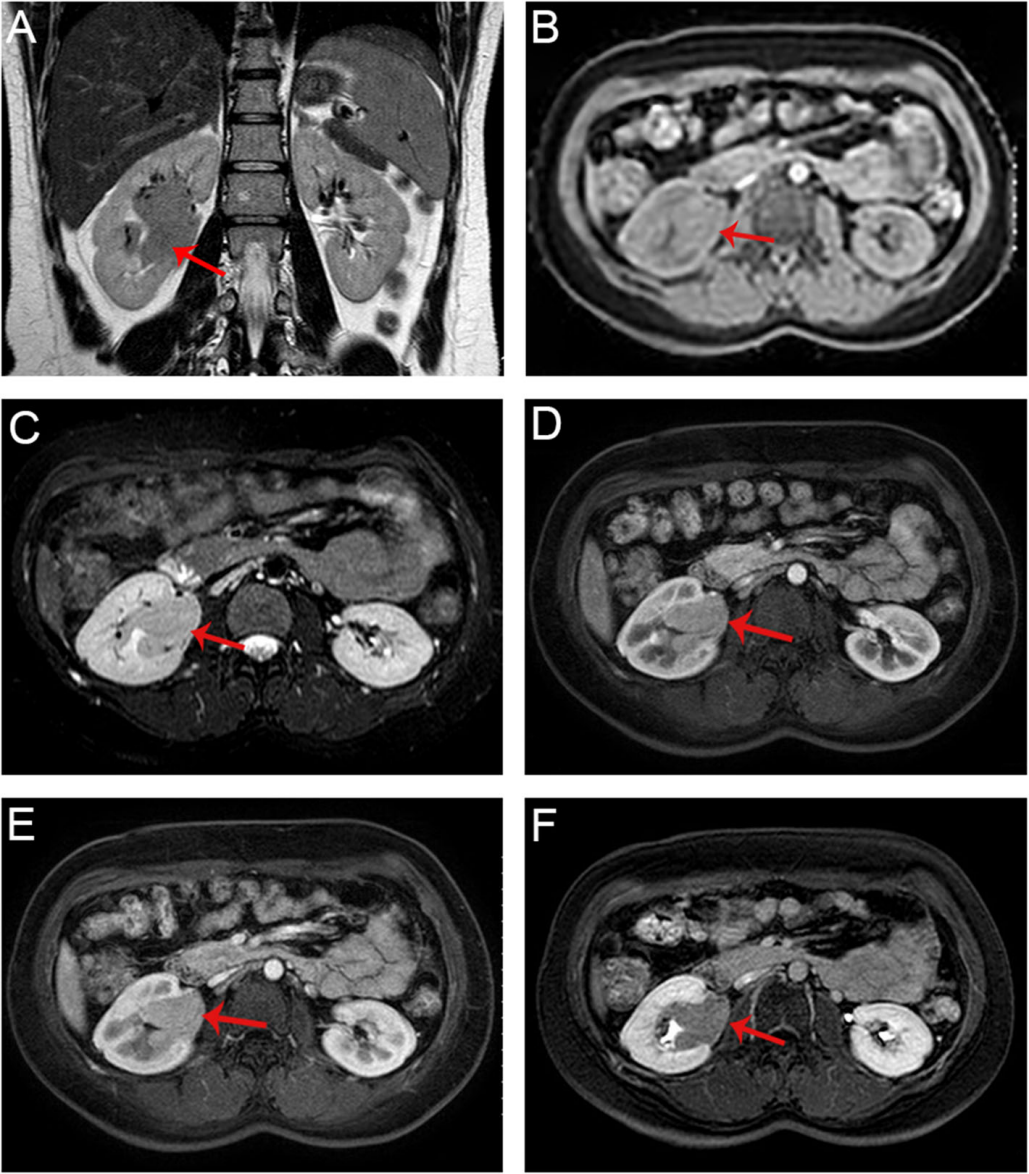
Contrast-enhanced abdominal MRI of the lesion (red arrow) in case 1. **(A)** Coronal T2-weighted image; **(B)** Transverse T1-weighted with fat suppression **(C)** Transverse T2-weighted with fat-suppression; **(D)** Transverse T1-weighted with fat-suppression in arterial phase; **(E)** Transverse T1-weighted with fat-suppression in portal phase; **(F)** Transverse T1-weighted with fat-suppression in delayed phase

A 32-year-old female was referred to our urology clinic and presented with persistent, painless gross hematuria and intermittent, blunt lumbago for 15 days. She did not report any fever, fatigue, edema, dysuria, urgent or frequent urination. Physical examination findings and all laboratory values, including complete blood count, renal and liver functions, and coagulation profile were unremarkable, except for urine erythrocyte at 343.5 /ul (normal range within 0–26 /ul). Urinary cytology and fluorescent in situ hybridization of exfoliated cells were both negative. However, contrast-enhanced abdominal MRI revealed an irregular mass, approximately 5.7 × 3.5 × 1.9 cm in size, in the right renal pelvis (Fig. 1A), with hypointensity on the T1 weighted image (Fig. 1B) and slight hyperintensity on the T2 weighted image (Fig. 1C). Meanwhile, the enhancement of the mass was markedly increased during the arterial phase (Fig. 1D) and decreased during the portal and delayed phases (Fig. 1E, F) with multiple enlarged lymph nodes (mild lymphadenectasis) in the right renal hilum. Based on the symptoms and imaging characteristics, a tentative diagnosis of carcinoma of the right renal pelvis was made.

**Fig. 2:**
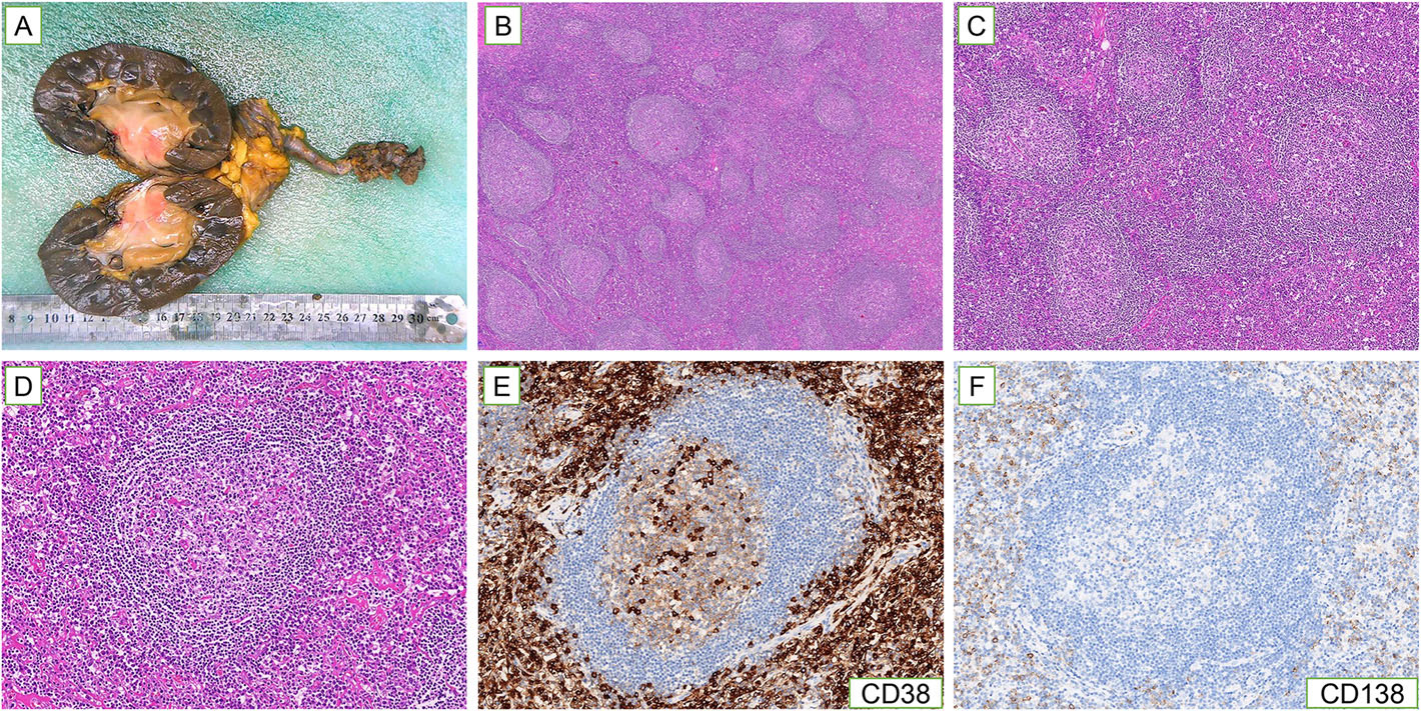
Histopathologic features of the mass in case 1. **(A)** Gross observation of the mass in the right renal pelvis. **(B)** Abundant lymphoid tissue with lymphatic follicles in the specimen (40×). **(C)** Numerous plasma cells infiltrate the paracortex zone and lymphatic sinus (100×). **(D)** Eosinophilic deposits were observed in the follicle centers (200×). **(E,F)** Immunohistochemistry showed that the interfollicular area was infiltrated by CD38 **(E)** positive and CD138 **(F)** positive plasma cells (200×)

Therefore, the patient underwent laparoscopic right radical nephroureterectomy with partial cystectomy and lymph node clearance around the renal pedicle. Intraoperatively, an elliptic solid tumor, approximately 6 × 4 cm in size, with unclear boundary, was located in the right renal pelvis. The cut surface appeared grayish-white in color, and sectioning showed hard texture without hemorrhage and necrosis. Grossly, the mass partially invaded the adipose tissue of the renal sinus, excluding the perirenal fat (Fig. 2A). Postoperative pathology reported lymphoid tissue hyperplasia in the specimen and eosinophilic deposits were observed in the follicle centers with numerous plasma cells infiltrating the paracortex zone and lymphatic sinus (Fig. 2B-D). Immunohistochemical staining indicated CD38 (+), CD138 (+) in the interfollicular plasma cells (Fig. 2E, F). In summary, the pathology and immunohistochemistry supported the diagnosis of PC type CD.

Subsequently, the patient visited the hematology department for further treatment of CD. A bone marrow biopsy was performed, which excluded hematological malignancies. Serology testing for human herpesvirus-8 (HHV-8) and human immunodeficiency virus (HIV) were both negative. A positron emission tomography with computed tomography (PET/CT) scan revealed slightly elevated metabolism of the operative region (SUV_max_ 2.7) and in multiple enlarged lymph nodes (SUV_max_ 2.4–3.3) including the parotid, neck, axilla, hilar and mediastinal nodes. Thereafter, she was treated with R-COP (rituximab 600 mg day1 + cyclophosphamide 1.2 g day2 + vindesine 4 mg day2 + prednisone 50 mg day2-day6) chemotherapy. Six courses later, the patient recovered, and symptoms disappeared. A follow-up PET/CT showed shrinkage and normal metabolism in the lymph nodes mentioned above with no recurrence.

### Case 2

A 45-year-old male with a history of hyperuricemia was brought to the local hospital with chief complaint of anuria for 2 days, with no occurrence of fever, edema, lumbago, hematuria, pyuria, or lower abdominal pain. CT of the abdomen indicated bilateral hydronephrosis and tumors in the renal pelvis and serum creatinine rose to 400 μmol/L (normal range within 32–116 μmol/L). Consequently, percutaneous ultrasound-guided nephrostomy was performed to relieve obstruction and an ultrasound-guided renal biopsy performed on both sides indicated lymphoma.

**Fig. 3:**
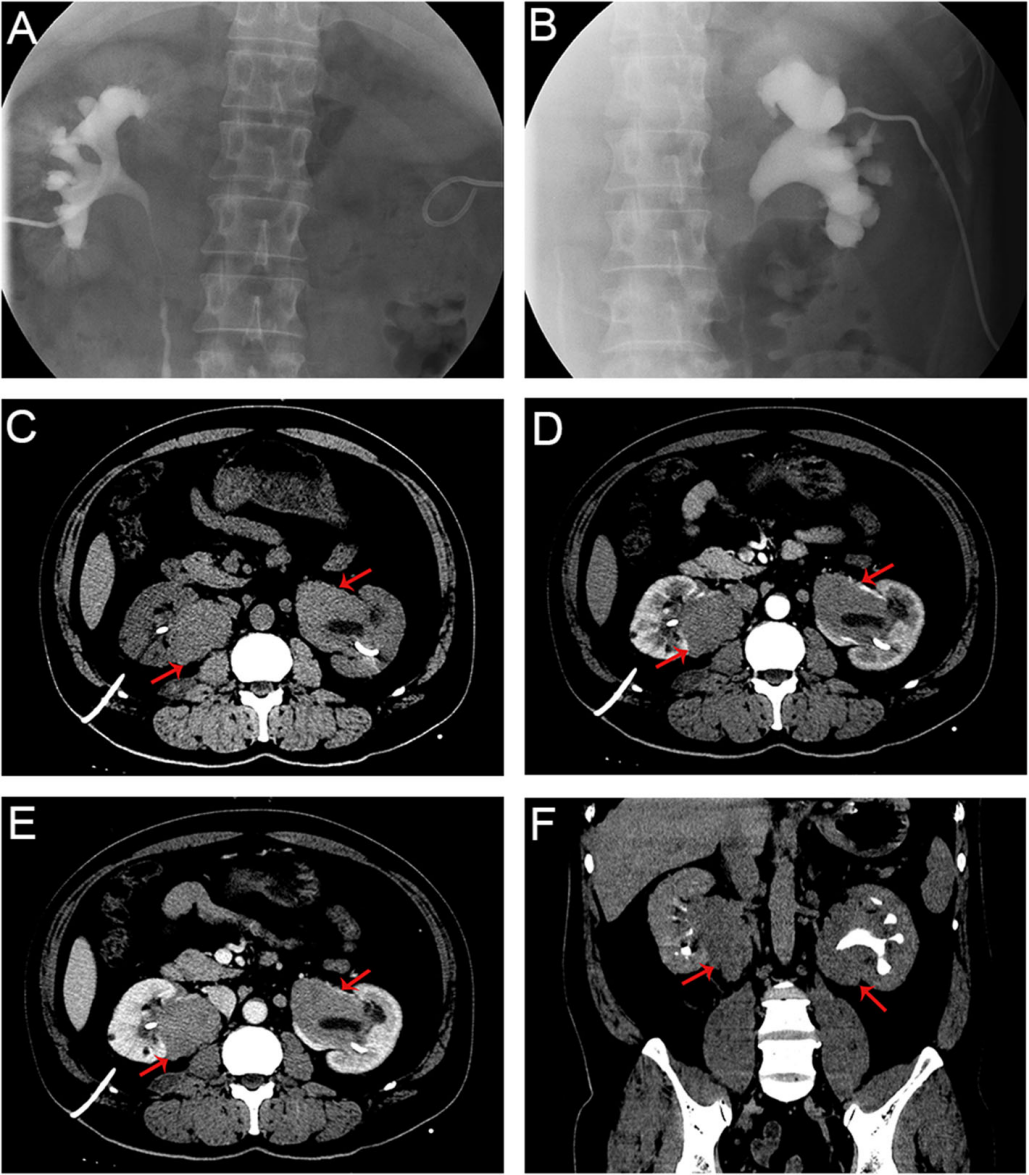
The antegrade urography of the left **(A)** and right **(B)** kidney, and contrast-enhanced abdominal CT urography of the lesions (red arrow) during plain scan phase **(C)**, arterial phase **(D)**, venous phase **(E)** and delayed phase **(F)** in case 2

A few days later he presented to our hospital for further treatment. The antegrade urography demonstrated bilateral hydronephrosis and ureteropelvic junction obstruction with prominent filling defect of the renal pelvis (Fig. 3A, B). Meanwhile, physical examination revealed no significant abnormalities. His laboratories were notable for low hemoglobin (113 g/L, with normal range within 130–175 g/L), high eosinophil count (0.67 * 10^9^/L, with normal range within 0.02–0.52 * 10^9^/L), high creatinine (146 μmol/L, with normal range within 32–116 μmol/L), and high uric acid (606 μmol/L, with normal range within 90–420 μmol/L). The glomerular filtration rate (GFR) of the left kidney was 14.5 ml/min, and that of the right was 22.2 ml/min. The subsequent contrast-enhanced CT urography (CTU) yielded soft-tissue density masses with an irregular border and mild-to-moderate homogeneous enhancement around the bilateral renal pelvis, measuring approximately 64 × 48 × 73 mm (right) and 74 × 50 × 74 mm (left). And the renal pelvis was compressed with a smooth surface. Obstruction at the ureteropelvic transition, with moderate dilatation and effusion of the renal pelvis and calyces was noticed. Multiple enlarged lymph nodes were observed in the lesser omental sac, hilum of liver and spleen, perirenal and paraaortic regions (Fig. 3C-F). Urine cytology did not reveal any cancer cells. Based on the above results, the patient was tentatively diagnosed with lymphoma preoperatively.

**Fig. 4:**
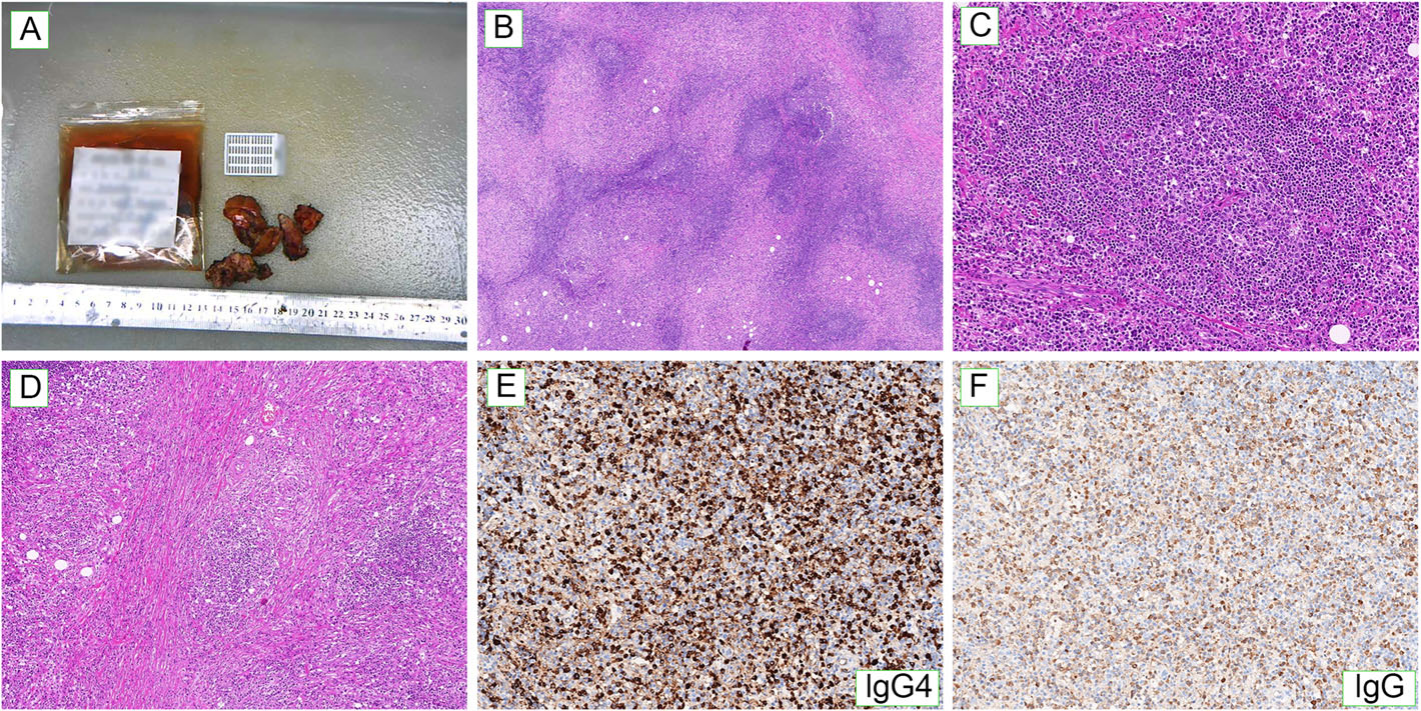
Histopathologic features of the masses in case 2. **(A)** Gross observation of resected masses. **(B)** Increased degree of fibrosis and dense infiltration of plasma cells and lymphocytes in the specimen (40×). **(C)** Plenty of lymphocytes and plasma cells infiltrate and form germinal centers (200×). **(D)** Storiform-type fibrosis and obliterative phlebitis (100×). **(E,F)** Immunohistochemistry revealed many IgG **(E)** and IgG4 **(F)** positive plasma cells (200×)

Intraoperative excisional biopsy and frozen section examination performed to identify the precise nature of the mass indicated a diagnosis of mixed-type CD and ruled out malignancy. To clinically remove the obstruction and rapidly improve renal function, the patient underwent partial resection of the renal hilum masses on both sides. On gross examination, the specimen was composed of a solid mass of gray-yellow and gray-red tissue measuring roughly 5 × 4 × 3 cm. Sectioning of the tissue revealed hard texture (Fig. 4A). Histopathological examination revealed increased degree of fibrosis and dense infiltration of plasma cells and lymphocytes, with storiform-type fibrosis and obliterative phlebitis (Fig. 4B-D). Immunohistochemistry demonstrated that many of the plasma cells were IgG (+) and IgG4 (+). More than 40% of the plasma cells were IgG4 (+) with more than 10 IgG4 (+) plasma cells per high powered field (HPF) of specimen (Fig. 4E, F). Serum IgG4 level was 27.40 g/L (normal range within 0.03–2.01 g/L). Thus, the diagnosis of IgG4-RD was finally confirmed, at which time treatment with methylprednisolone (40 mg/day) and cyclophosphamide (0.2 g every other day) was initiated, followed by a gradual decrease of the dose. Repeat CT scan 3 months later revealed no hydronephrosis, with significant reductions in the size of renal lesions and enlarged lymph nodes. Follow-up studies performed 9 months after the diagnosis confirmed a decrease in serum IgG4 and creatinine to 2.21 g/L and 118 μmol/L, respectively. No recurrence was observed as the patient was prescribed low dose methylprednisolone.

## Discussion and conclusions

Our cases highlighted the imaging of renal space-occupying lesions and the symptoms related to presence of a renal mass, including painless gross hematuria, lumbago, and anuria. None of these symptoms directly indicate CD and IgG4-RD. However, these symptoms and imaging findings often raise high suspicion of kidney malignancy, frequently leading to misdiagnosis because of its increasing frequency and higher risk [9]. The widespread availability of abdominal CT, MRI, and ultrasound has led to an increase in the diagnosis of renal tumors. In 2018, there were more than 400,000 new cases of cancers of the kidney and renal pelvis globally, representing an estimated 2.2% of the global cancer burden, with more than 175,000 deaths due to this disease [10].

The diagnosis and management of kidney cancer have developed rapidly in recent years. The symptoms of kidney cancer include the classic clinical triad of hematuria, flank pain and palpable mass, as well as paraneoplastic syndromes [11, 12]. However, over 50% of renal masses are detected coincidentally, with only a few cases presenting with the classical symptoms mentioned above. Furthermore, the presence of these symptoms is often indicative of locally advanced or metastatic kidney cancer [13]. Imaging tests and renal biopsy, as well as laboratory tests like urinary cytology, are crucial methods to diagnose kidney malignancy [14]. However, imaging evaluation may sometimes lead to misdiagnosis when encountering rare diseases such as CD and IgG4-RD. They can not only pose a diagnostic challenge since these lesions masquerade as kidney malignancy, but also result in unnecessary radical nephrectomy, and delay appropriate treatment [15, 16]. In fact, percutaneous needle core biopsy has been increasingly applied to guide clinical decisions in the management of renal masses, not only for its relatively high accuracy and safety in evaluating suspicious and undefinable masses, but also for avoiding unnecessary surgery [17–19]. Additionally, it is important to recognize that patients managing with nephrectomy, especially radical nephrectomy, have a high chance of developing renal insufficiency [20]. Impairment of kidney function after nephrectomy may eventually lead to chronic kidney disease. This is even more likely in patients who show a decline in GFR of the other kidney before the surgery [21]. Thus, distinguishing these two diseases from kidney malignancy is urgently required.

CD is an atypical and rare lymphoproliferative disease originally described by Benjamin Castleman in 1956 [22]. The incidence rate of CD is roughly 21–25 per million person-years [23]. CD can manifest at any age with the peak age of onset between 30 and 50 years, without significant gender differences [24]. The role of HHV-8 and HIV in the pathogenesis of CD is well documented [25, 26]. However, there was no evidence for HHV-8 and HIV infection in our patient. Clinically, CD can be divided into two subtypes: unicentric CD (UCD) and multicentric CD (MCD), based on its involvement in a single lymph node or region of lymph nodes and multiple lymph nodes, respectively. UCD patients are typically diagnosed in those with compressive symptoms or enlarged lymph nodes that are found serendipitously. Most of them are asymptomatic with normal laboratory findings. In contrast, MCD patients often present with lymphadenopathy in more than a single lymph node station accompanied by fever, anemia, emaciation, liver and kidney dysfunction, and other systemic manifestations, including TAFRO syndrome (thrombocytopenia, ascites, reticulin fibrosis, renal dysfunction, and organomegaly) [27, 28]. Our PET/CT scan of the patient suggested MCD with the median SUV_max_ between 2.4 and 3.3. Regardless of the type of CD, the median SUV_max_ is usually approximately 3–8, whereas higher values would be suggestive of malignancy [29]. However, in our case, patient mainly presented with hematuria and lower back pain, whereupon preoperative MRI indicated occupying lesion in the renal pelvis. Due to the lack of specificity in imaging findings, it is difficult to distinguish them from kidney malignancy by imaging alone. Furthermore, the postoperative histopathological result of CD is generally different from the preoperative imaging diagnosis [30]. Unfortunately, core needle biopsy was not taken to confirm the preoperative pathological diagnosis, resulting in subsequent radical nephrectomy in case 1.

In fact, the diagnosis of CD depends entirely on histopathology. Histologically, CD can be divided into three subtypes, namely, HV, PC an intermediate mixed type. The HV and PC types account for approximately 90 and 10% of CD, respectively, whereas the mixed type is rare [31]. The pathology of the HV type is characterized by the proliferation of interfollicular capillaries and small hyaline vascular follicles. In contrast, PC type mainly manifests as the spreading of sheets of mature plasma cells in the interfollicular areas and hyperplastic germinal centers. The mixed type has morphological features of both types [4, 32]. Pathologically, the CD patient in our report was finally diagnosed as PC type with no significant systemic symptoms. The interfollicular zone was infiltrated by CD38 (+) and CD138 (+) plasma cells and amorphous eosinophilic deposition was observed in the germinal centers.

Generally, the treatment of UCD primarily based on surgical resection. Complete resection of involved lymphoid tissue is relatively recognized as the gold standard for management and can eliminate clinical symptoms with low recurrence [33]. Ten-year overall survival rate in UCD patients is approximately 90% [24]. Moreover, radiation therapy can be considered in those unsuitable for surgery [33]. Contrastingly, surgery is insufficient for the management of MCD with lower overall survival rate than UCD [24, 34]. The management of MCD is mainly based on glucocorticoids, chemotherapy, antiretroviral therapy, and monoclonal antibody therapy targeting IL-6 [33]. In case 1, after radical nephroureterectomy performed due to the misdiagnosis of CD as carcinoma of the renal pelvis, the patient underwent combination chemotherapy (R-COP) for the postoperative pathological diagnosis of CD. Fortunately, no recurrence was observed in the follow-up PET/CT after chemotherapy.

IgG4-RD is a newly recognized chronic and progressive autoimmune disorder of unclear etiology, involving multiple organs and tissues. The disease responds well to glucocorticoids. It is characterized by elevated serum IgG4 concentrations and tumefactive lesions in the affected organs, caused by abundant infiltration of IgG4-positive plasma cells and lymphocytes with fibrosis [3, 35]. The epidemiology of the disease remains poorly reported due to lack of recognition. However, Japan has estimated that the incidence of this disease would be 0.28–1.08/100,000 population per year with the mean age of affected patients being 60 to 70 years [6]. IgG4-RD exhibits more common in males with a male-to-female ratio of 2 to 1 [36]. Clinical symptoms of IgG4-RD vary depending on the involved organs, but most patients have relatively mild symptoms with a long disease course. Furthermore, IgG4-RD is likely to go undiagnosed or misdiagnosed because it is treated in different specialties depending on organ involvement [37]. In case 2, our patient was sent to the urology department for anuria. Unlike other autoimmune diseases, systemic symptoms such as fever, malaise, swelling, and pain in joints are uncommon [38]. Hypergammaglobulinemia is frequently observed in laboratory testing of patients with IgG4-RD. Serology often reveals elevation in IgG, IgE, and eosinophil count [39]. Serum IgG4 > 135 mg/dl (1.35 g/l) is the suggested cutoff value or diagnosis of IgG4-RD, with a sensitivity and specificity of 97.0 and 79.6%, respectively [40]. However, we did not consider IgG4-RD as a diagnosis before surgery, and consequently, serum IgG4 concentration was not evaluated preoperatively. We then considered the mass lesions as lymphoma in CTU. Imaging of IgG4-RD patients usually reveals enlargement or compression of the different organs involved. Contrast-enhanced CT scans of IgG4-RD with kidney involvement often presents multiple well demarcated low-density lesions in the kidney or thickening of the renal pelvis wall [37]. CTU in case 2 revealed large soft tissue lesions, but with mild-to-moderate homogeneous enhancement and smooth renal pelvis surface, which is a noteworthy imaging feature for urologists to identify kidney malignancy.

The diagnosis of IgG4-RD is primarily based on histopathological examination. Specific features include dense lymphoplasmacytic infiltrates in affected tissues or organs, obliterative phlebitis and storiform fibrosis [41]. Additionally, to diagnose IgG4-RD, the ratio of IgG4-positive plasma cells to IgG-positive plasma cells should be greater than 40% in the affected tissue, with a sensitivity of 94.4% and a specificity of 85.7% [40]. However, increased IgG4-positive plasma cells is not a specific feature of IgG4-RD and can exhibited in various chronic inflammatory diseases like inflammatory bowel disease, vasculitis, and lymphoma, which do not exhibit histological features of storiform-type fibrosis and obliterative phlebitis [36]. In case 2, CD was diagnosed by intraoperative frozen section examination, but paraffin-embedded pathology and immunohistochemistry after surgery confirmed IgG4-RD. MCD is often difficult to distinguish from IgG4-RD since they have overlapping clinical and/or histological features [42]. In 2011, the comprehensive clinical diagnostic criteria for diagnosis of IgG4-RD were established in Boston [43], which include [1] clinical manifestations of characteristic diffuse/localized enlargement or mass formation in single or multiple organs, [2] Serological tests, presenting elevated serum IgG4 levels ≥135 mg/dl, and [3] histopathological features, showing dense infiltration of lymphocytes and plasma cells with fibrosis, and the ratio of IgG4-positive plasma cell / IgG-positive plasma cell > 40% and > 10 IgG4-positive plasma cells per HPF. Only when all three criteria are met can a definitive diagnosis be made. A probable diagnosis can be made if conditions 1 and 3 are met, and 1 and 2 can make a possible diagnosis. In case 2, we made a definitive diagnosis of IgG4-RD based on all three criteria above after surgery.

Once we confirm the diagnosis of IgG4-RD, glucocorticoids, a well-recognized therapy, can be considered for patients. Patients treated with glucocorticoids have an overall response rate and complete response rate of 93 and 66%, respectively [36]. Combined use of immunosuppressants such as cyclophosphamide, mycophenolate mofetil, and azathioprine may be effective in the maintenance of remission, allowing for the reduction of steroids while helping prevent recurrence. In patients exhibiting glucocorticoid resistance or relapsing disease, rituximab is a better alternative [36, 44]. Moreover, urgent surgical intervention is needed to reduce the severity of obstruction symptoms and improve organ functions rapidly [44]. Our patient in case 2 responded well to glucocorticoids and cyclophosphamide after partial excision of the mass lesion. The follow-up imaging tests showed that drug therapy caused continued reduction in the size of the mass, indicating that the surgical excision may be unnecessary. Hence, we need to consider whether surgery for IgG4-RD can be avoided to reduce surgical risk and financial costs associated with overtreatment of benign conditions.

In conclusion, we reported two cases of two rare diseases, CD and IgG4-RD, presenting with renal space-occupying lesions and masquerading as kidney malignancy. The prominent feature in our cases is that preoperative diagnosis was difficult since CD and IgG4-RD can exhibit nonspecific clinical manifestations and imaging findings, and our diagnosis was eventually based on postoperative pathology. Our cases highlight how CD and IgG4-RD can affect the kidney, manifest as mass lesion, and mimic malignancy. Therefore, we recommend that clinicians should perform core biopsy while diagnosing patients with suspicious and undefinable renal masses to avoid unnecessary surgical treatment.

## Data Availability

Data are available on reasonable request from the corresponding author due to privacy or other restrictions.

## Abbreviations

CD: Castleman disease
IgG4-RD: IgG4-related disease
CT: computed tomography
MRI: magnetic resonance imaging
HV: hyaline vascular
PC: plasma cell
PET/CT: positron emission tomography with computed tomography
R-COP: R = Rituximab
C: Cyclophosphamide
O: Vincristine (Oncovin)
P: Prednisone
GFR: glomerular filtration rate
CTU: computed tomography urography
HPF: high powered field
HHV-8: human herpesvirus-8
HIV: human immunodeficiency virus
UCD: unicentric Castleman disease
MCD: multicentric Castleman disease
